# Systematic Evaluation of the Immune Environment of Small Intestinal Neuroendocrine Tumors

**DOI:** 10.1158/1078-0432.CCR-21-4203

**Published:** 2022-03-23

**Authors:** Clare Vesely, Yien Ning Sophia Wong, Alexa Childs, Ayse U. Akarca, Pawan Dhami, Heli Vaikkinen, Lucia Conde, Javier Herrero, Olagunju Ogunbiyi, Amir Gander, Tu Vinh Luong, Chrissie Thirlwell, Martyn Caplin, Christos Toumpanakis, Karl Peggs, Sergio A. Quezada, Teresa Marafioti, Tim Meyer

**Affiliations:** 1 UCL Cancer Institute, UCL, London, United Kingdom.; 2 Cancer Immunology Unit, Research Department of Hematology, UCL Cancer Institute, UCL, London, United Kingdom.; 3 Royal Free Hospital, Pond Street, London, United Kingdom.; 4 The University of Exeter Medical School, Exeter, United Kingdom.

## Abstract

**Purpose:**

The immune tumor microenvironment and the potential therapeutic opportunities for immunotherapy in small intestinal neuroendocrine tumors (siNET) have not been fully defined.

**Experimental Design:**

Herein, we studied 40 patients with primary and synchronous metastatic siNETs, and matched blood and normal tissue obtained during surgery. We interrogated the immune checkpoint landscape using multi-parametric flow cytometry. In addition, matched FFPE tissue was obtained for multi-parametric IHC to determine the relative abundance and distribution of T-cell infiltrate. Tumor mutational burden (TMB) was also assessed and correlated with immune infiltration.

**Results:**

Effector tumor-infiltrating lymphocytes (TIL) had a higher expression of PD-1 in the tumor microenvironment compared with the periphery. In addition, CD8^+^ TILs had a significantly higher co-expression of PD-1/ICOS and PD-1/CTLA-4 (cytotoxic T lymphocyte antigen-4) and higher levels of PD-1 expression compared with normal tissue. IHC revealed that the majority of cases have ≤10% intra-tumoral T cells but a higher number of peri-tumoral T cells, demonstrating an “exclusion” phenotype. Finally, we confirmed that siNETs have a low TMB compared with other tumor types in the TCGA database but did not find a correlation between TMB and CD8/Treg ratio.

**Conclusions:**

Taken together, these results suggest that a combination therapy approach will be required to enhance the immune response, using PD-1 as a checkpoint immunomodulator backbone in combination with other checkpoint targeting molecules (CTLA-4 or ICOS), or with drugs targeting other pathways to recruit “excluded” T cells into the tumor microenvironment to treat patients with siNETs.

**Translational Relevance**

Most patients with small intestinal neuroendocrine tumors (siNET) present with advanced, metastatic disease. Current standard therapies consistently offer objective response rates of less than 20% and trials of immune checkpoint inhibitors to date, have failed to improve on this. Here, we have performed a detailed analysis of the immune microenvironment in siNET to inform a rational approach for targeting the immune system in this tumor. We demonstrated that effector tumor-infiltrating lymphocytes had a significantly higher expression of checkpoint molecules, including high levels of PD-1 expression in the tumor microenvironment compared with the periphery and that PD-1 is frequently co-expressed with other checkpoint molecules, including cytotoxic T lymphocyte antigen-4 and ICOS. Moreover, IHC revealed an exclusion immune phenotype with a higher number of T cells peri-tumorally compared with intra-tumorally. These findings suggest that combination therapy will be required to target multiple checkpoints and strategies to reverse the immune exclusion phenotype need to be explored.

## Introduction

An adaptive immune response is initiated through antigen recognition by a T-cell receptor (TCR) on a T cell, but the final quality and amplitude of the immune response elicited is ultimately dictated by an additional layer of checkpoint signaling (1). This signaling is regulated through a balance of co-inhibitory and co-stimulatory checkpoint molecules found on the cell surface. These immune checkpoints are needed under normal physiological conditions in preventing autoimmunity in the form of “self-tolerance.” One mechanism by which tumors evade immune surveillance is by dysregulation of these immune checkpoint molecules. There is now a myriad of immune checkpoint inhibitors (CPI) in various stages of clinical development that acts by targeting checkpoint molecules, to tip the immune response back toward tumor destruction (1–3).

This approach has been effectively used to target the checkpoint molecule programmed cell death-1 (PD-1) and its ligand, programmed cell death ligand-1 (PD-L1) with therapeutic antibodies, including nivolumab, pembrolizumab, atezolizumab, and durvalumab, in a range of advanced solid malignancies (4–6). Moreover, the mAb, ipilimumab, which targets the cytotoxic T lymphocyte antigen-4 (CTLA-4) is the first drug in 30 years to improve survival in metastatic melanoma, a disease with previously poor survival outcomes and minimal treatment options (7). Critically, these patients with metastatic melanoma who respond can derive long-term, durable remissions, particularly to combination CPIs that target different checkpoints (8).

Neuroendocrine tumors are a diverse group of neoplasms that arise from enterochromaffin cells located throughout the body, but most commonly within the gastrointestinal (GI) tract, pancreas, and lungs. They are remarkable for their heterogeneity in terms of biology, clinical behavior and prognosis, with a median survival of approximately 10 months in patients with metastatic high-grade tumors, as compared with 16.2 years in low-grade disease (9). Not only do the anatomical subgroups have distinct molecular profiles, but they also respond differently to therapies, including cytotoxic chemotherapy, receptor tyrosine kinase inhibitors, and CPIs. For this reason, and to reduce the impact of confounding factors, we confined our analysis to a well-defined anatomical subgroup.

The majority of patients with small intestinal neuroendocrine tumors (siNET) present with advanced metastatic disease that is not amenable to curative surgery, and a range of systemic therapies may be offered, including somatostatin analogues (SSA; 10), cytotoxic chemotherapy (11), the mTOR inhibitor everolimus (12), and peptide receptor radionuclide therapy (PRRT; ref. 13). Objective response rates to these treatments are consistently less than 20% and there is a need to develop more effective therapies with more sustained tumor control rates. Immunotherapeutic approaches have been successfully applied to high-grade neuroendocrine tumors, including Merkel cell carcinoma, and both avelumab and pembrolizumab have been approved in this indication. However, efficacy data from small cohorts of GI NET have been disappointing with 2% response rate reported for pembrolizumab (14), 3.1% for spartalizumab (15, 16), and no responses reported for durvalumab and tremilimumab. The majority of GI NETs are low-grade and data from the combination of ipilimumab plus nivolumab demonstrate better response rates in high-grade tumors at 44% compared with 0% in low-grade tumors (17).

Although there have been a number of studies that have evaluated PD-1 or PD-L1 expression in siNETs (18), a comprehensive analysis of the immune checkpoint profile in siNET has not been reported. We hypothesize that a deeper understanding of the molecular landscape might provide a rationale for the development of immunotherapy in siNET and in this study have performed a systematic analysis of the immune microenvironment in 40 patients with siNET.

## Materials and Methods

### Patients and samples

Eligible patients were required to have histologically confirmed NET of small intestinal origin. Data were collected on age, gender, primary site, and grade according to the European Neuroendocrine Tumor Society (ENETS)/WHO guidelines (19). This study was conducted in accordance with the Declaration of Helsinki, was approved by the Local Ethics Committee (IRAS REC reference 15/LO/1567), and all participants were required to provide written informed consent. Peripheral blood samples were collected into a 10-mL EDTA tube. Peripheral blood mononuclear cells (PBMC) were isolated by density-gradient centrifugation with Ficoll–Paque PLUS (GE Healthcare). Isolated live cells were frozen at −80°C and stored until analysis. Fresh tissue samples were dissected surgically, and both tumor regions and normal adjacent were defined macroscopically by a pathologist and collected in serum-free RPMI culture medium. Single-cell suspensions were prepared by mechanical disruption of tissue using a scalpel and digestion with a mixture of 0.2 mg/mL DNase and 0.3 mg/mL Liberase TL or 2.5 mg/mL Collagenase in serum-free RPMI for 1 hour on a GentleMACS Dissociator (Miltenyi Biotec). Samples were filtered through a 70-µm cell strainer, and leukocytes were enriched by density-gradient centrifugation with Ficoll-Paque PLUS (GE Healthcare). Finally, the isolated cells were frozen at −80°C for 24 hours and then cryogenically stored until analysis.

### Flow cytometry

Acquisition was performed with a BD LSR II Fortessa (BD Biosciences). The following antibodies and fluorescent labels were used to stain samples for T-cell analysis: From BioLegend: PD-1-BV605 (EH12.2H7), CD3-BV785 (OKT3), CTLA-4–APC (L3D10), ICOS-PE/Cy7 (C398.4A), OX40-PE/Cy7 (Ber-ACT35), streptavidin-APC, and 4–1BB-PE (4B4–1); from BD Biosciences: CD8-V500 (SK1), and TIM-3-BV650 (7D3); from eBioscience: FoxP3-PerCP-Cy5.5 (PCH101), CD4-AF700 (OKT-4), TIGIT-PE (MBSA43), and fixable viability dye-e780; and GITR-Biotin (DT5D3) from Miltenyi Biotec. Intranuclear staining of FoxP3 and Ki67 was performed using the FoxP3/Transcription Factor Staining Buffer Set (eBioscience) and FITC Mouse Anti–Ki-67 Set (BD Biosciences). Data were analyzed with FlowJo v.10.6.0 (BD Biosciences) and samples were required to have >20 cells in all subsets to be eligible for analysis. For co-expression data, samples with >500 viable CD3 cells were taken forward for analysis. Samples were concatenated and analyzed using FlowJo plugins (https://flowjo.com/exchange/#/), namely: Downsample (v.3.2), UMAP (uniform manifold approximation and projection; v2.2), and FlowSOM (v2.6).

### Multiplex IHC

Three-μm tissue sections were cut and transferred onto poly-l-lysine–coated slides, dewaxed in two changes of xylene, and rehydrated in a series of graded alcohols. Sections were stained with hematoxylin and eosin according to standard procedures. Primary antibodies used were anti-CD4 [clone: SP35; dilution: 1/50 (3.1 µg/mL); Abcam Plc.; ref. 20], anti-CD8 [clone: SP239; dilution: 1/100 (5.0 µg/mL); Abcam Plc.], anti-FOXP3 [clone: 236A/E7; dilution: 1/100 (99.8 µg/mL]; a kind gift from Dr. G. Roncador (CNIO, Madrid, Spain; refs. 21, 22); and anti-Cytokeratin [clone: AE1/AE3; dilution: 1/100 (17.1 µg/mL); Agilent Technologies Inc.]. Single IHC was carried out using the automated platforms BenchMark Ultra (Ventana/Roche) and the Bond-III Autostainer (Leica Microsystems) according to a protocol described elsewhere (refs. 23, 24; Supplementary Fig. S1). To establish optimal staining conditions (i.e., antibody dilution and incubation time, antigen retrieval protocols, suitable chromogen) each antibody was tested and optimized on sections of human reactive tonsil, used as positive control. Multiplex IHC was carried out using a protocol described previously (25). Briefly, pre-treated sections were incubated 30 minutes with the first primary antibody at room temperature and then developed with peroxidase-based detection system (Envision System; Agilent Technologies, Inc.) to visualize antibody-binding site. The following step included the second antibody incubation, and its visualization with an alkaline phosphatase kit (Dako REAL Detection System; Agilent Technologies, Inc.). Finally, both steps were repeated for the application of third and fourth antibodies. After staining, samples were washed in buffers and distilled water and mounted in Apathys mounting medium (TCS Biosciences Ltd.). Specificity of the staining was assessed by a hematopathologist (T. Marafioti) according to the conventional principles of antibodies validation measuring the specificity of the protein cellular localization; the sensitivity of the antibody staining was controlled by using different antibodies concentration. Slides were scanned using the NanoZoomer Digital Pathology System C9600 (Hamamatsu).

### IHC analysis

Immune cells from scanned images were analyzed using QuPath 0.1.2 (open-source software, ref. 26). The T-cell analysis strategy was designed to approximate recommendations made by Obeid and colleagues (27) to sample multiple small areas encompassing 3 mm^2^ within the central tumor avoiding areas of dense lymphoid aggregates (DLA) and away from the periphery and invasive margin. For this reason, we selected six central tumor regions of 500 µm^2^ avoiding areas of DLA to encompass a total area of 3 mm^2^. Cytokeratin was used as tumor marker to identify the tumor area. Each case was scored first to assess the distribution of the inflammatory cells within the intra- and peri-tumoral area and we selected six regions as representative of the trend of immune cell distribution. Where multiple lesion sites were present, we spread our sampling areas between the major sites to as best as possible represent the whole tumor. The number of cells were manually counted using NDP.view2 viewing software (Hamamatsu).

### Statistical analysis

Statistical analyses were done with GraphPad Prism 7.03 (GraphPad Software); *P* values were calculated using the Kruskal–Wallis analysis of variance and Dunn's *post hoc* test with error bars represent mean values with SEM, unless otherwise indicated (ns = *P* > 0.05; *, *P* < 0.05; **, *P* < 0.01; ***, *P* < 0.001; ****, *P* < 0.0001).

### Genomic DNA extraction

Ninety-six samples from 40 patients, including 56 formalin-fixed paraffin-embedded (FFPE) tumor tissue tumors and 40 matching controls (9 FFPEs, 31 fresh-frozen) were sent for exome sequencing. DNA was extracted from 10-µm sections of FFPE tumor tissue using the DNAstorm FFPE DNA Isolation Kit (CELLDATA) according to the manufacturers’ instructions. Matched germline DNA was extracted from 1-mL peripheral blood using the DNeasy Blood and Tissue Kit (QIAGEN) following the manufacturers’ protocol. DNA concentrations were measured using the NanoDrop-1000 Spectrophotometer (NanoDrop) and Qubit 2.0 Fluorometer (Invitrogen).

### Exome-seq library preparation and sequencing

DNA samples were quantified using either Qubit dsDNA high sensitivity or Qubit dsDNA Broad Range kit (Invitrogen). DNA quality was assessed, and a DNA-integrity number (DIN) assigned using Agilent Tapestation (Agilent). Exome-Seq libraries for the samples were generated using Roche SeqCap EZ HyperCap Workflow, using KAPA HyperPlus library preparation kit. One hundred and fifty ng of genomic DNA for fresh-frozen, 300 ng for FFPE samples (DIN > 3), and 1,000 ng for FFPE samples (DIN < 3) were used as starting input for library preparation.

AMPure XP beads (Beckman Coulter) were used to perform double-sided size selection of the libraries according to the Roche SeqCap EZ HyperCap workflow (Roche). Libraries were assessed for average fragment size and quality using Agilent Bioanalyzer DNA 1,000 chips and quantified using KAPA Library quantification kit for Illumina platforms (Roche). Roche SeqCap EZ MedExome Enrichment kit and exome panel were used according to Roche SeqCap EZ HyperCap workflow to perform exome capture.

Post amplification, the Exome-Seq libraries were assessed for average insert size using Agilent Bioanalyzer DNA 1,000 chips and quantified using KAPA Library quantification kit for Illumina platforms (Roche).The molar concentration of the libraries was determined using the following formula:




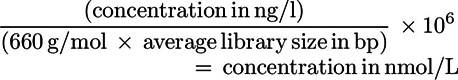




The Exome-Seq libraries were manually normalized to 10 nmol/L and combined to generate eight library pools that were sequenced on Illumina HiSeq 2500 platform, using two High-Output flow cells, with cluster generation performed on Illumina cBot instrument to obtain the required number of reads needed.

### Genomic analysis

FastQ files were processed with the SciLifeLab/Sarek v2.3.FIX1 pipeline (28). Specifically, reads were aligned against GRCh38 using BWA v0.7.17 and processed using GATK v4.1.1.0. The coverage in tumor samples ranged from 36X to 142X (median 74X) and in controls from 25X to 76X (median 54X). Somatic variant calling was performed with mutect2 following the GATK (v4.1.9.0) best practices, using a panel of normal created from the 40 control samples used in the analysis, and with the mutect2—tumor-lod-to-emit argument (TLOD threshold) set to 7.3. The funcotator tool from GATK v4.1.9.0 was used to annotate the variants according to their effect on the canonical transcript, and the maftools R package was used to create mutation summary plots.

Initial analysis pointed to an obvious excess of C>T mutations, consistent with known artefacts in FFPE samples resulting from deamination during formalin fixation. To decrease the amount of C>T false positives and improve the quality of the callset, we used a higher TLOD threshold for C>T mutations and filtered out C>T mutations of low allele frequency, common gnomad variants (AF>0.0001), low-confidence variants (n_alt_count > 1 | t_depth < 30 | t_alt_count ⇐ 5), and variants in the top-20 Frequently Mutated Genes (FLAGS; ref. 29).

### Data availability statement

The data generated in this study are available within the article and its Supplementary Data Files.

## Results

### Patient cohort

Fresh tissue and blood were collected from 40 patients with a confirmed diagnosis of siNET from the Royal Free Hospital (London, UK) between September 2015 and June 2018. Thirty-nine samples were collected from patients undergoing surgical resections and one was collected from a patient undergoing a tissue biopsy. The median age was 62 with range, 27–85 years. Metastatic disease was present at the time of the procedure in 75% (*n* = 30). Overall, 65% (*n* = 26) had grade 1 disease, 32.5% (*n* = 13) grade 2, and 2.5% (*n* = 1) grade 3. At the time of sample collection, 60% (*n* = 24) of patients were receiving SSAs and one had undergone PRRT; the rest were treatment naïve (Table 1). In each case, fresh tissue was collected from the tumor (*n* = 25) and, for those undergoing surgical resection, normal control tissue was also sampled from a distal part of the resection specimen where possible (*n* = 22). Normal tissue was sampled at least 5 cm from the macroscopically visible tumor by an experienced pathologist. Metastatic tumor was also sampled where feasible (*n* = 23). Peripheral blood was collected concurrently at time of surgery: one sample for genomic DNA extraction and a second for immune cell profiling. FFPE tissue was also obtained for IHC and tumor genomic DNA extraction.

**Table 1.** Clinicopathological features of siNET cohort.

	Number	(%)
Total	40	
Sex		
Male	23	57.5%
Female	17	42.5%
Age, median (range)	62	(27–85)
Metastatic site	30	75.0%
Ki-67–labeling index		
≤2% (Grade 1)	26	65.0%
>2%–20% (Grade 2)	13	32.5%
>20% (Grade 3)	1	2.5%
Treatment		
Nil	15	37.5%
SSA	24	60.0%
PRRT	1	2.5%

Abbreviations: Nil, Patient not on any treatment before sample collection; PRRT, Peptide Receptor Radionuclide Therapy; SSA, somatostatin analogues.

### Evaluation of T-cell subsets indicates that siNET tumors have a lower effector T cells to regulatory T cells ratio than normal tissue in CD8 and PBMC in CD4eff

**Figure 1.**
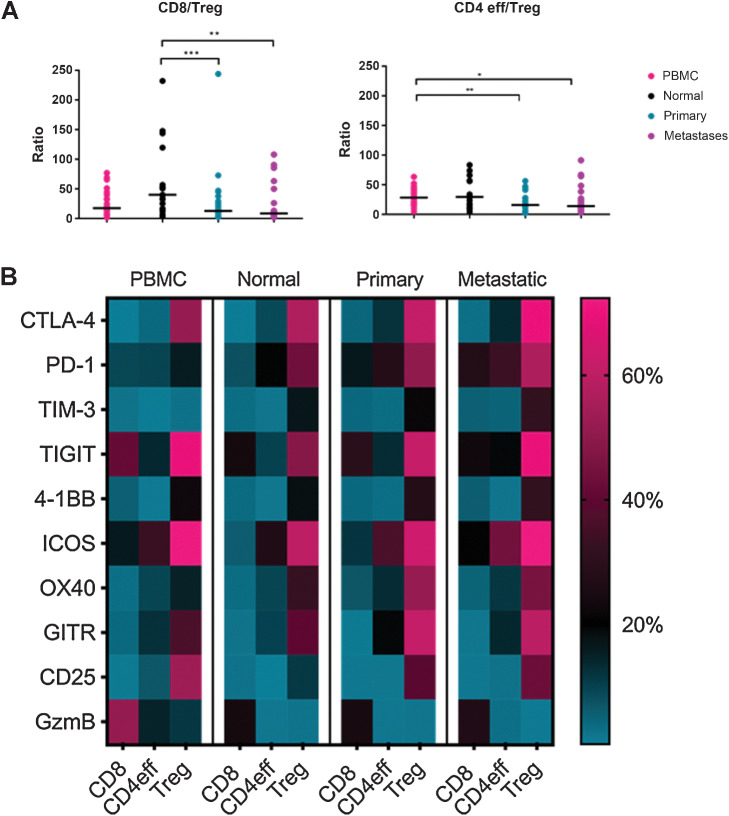
Effector/Treg ratio is significantly lower in tumor tissues and CD8^+^ in metastatic tissue is significantly more regulated compared with other tissue types. **A,** The horizontal black bars indicate the median values for each compartment. The ratio of effectors to regulatory T cells was significantly lower in primary or metastatic tumor tissues compared with the normal tissue (CD8/Treg) and PBMCs (CD4eff/Treg). **B,** Single level expression and co-expression of B7 and TNFR superfamily co-inhibitory and co-stimulatory molecules on T-cell subsets were quantified by flow cytometry in matched peripheral blood mononuclear cells (PBMC), normal tissue, primary, and metastatic tumor tissues obtained from all patients. Displayed is a heatmap depicting the mean percentage of CD8^+^, CD4eff (CD4^+^ FoxP3^–^), and Treg (CD4^+^ FoxP3^+^) cells expressing individual immune checkpoint molecules and proliferating markers in each tissue sample. *, *P* < 0.05; **, *P* < 0.005; ***, *P* < 0.0005.

Isolated lymphocytes were analyzed by flow cytometry to define T-cell (CD3^+^) subpopulations: CD8^+^ cytotoxic T cells, CD4^+^ FoxP3^–^ effector T cells (CD4eff), and CD4^+^ FoxP3^+^ regulatory T cells (Treg; Supplementary Fig. S2). The samples included PBMCs (*n* = 32), normal bowel tissue (*n* = 35), primary tumor tissue (*n* = 32), and metastases [consisting mainly of mesenteric masses (*n* = 23) or metastatic lymph nodes (*n* = 2)]. The ratio of the effector T cells to Tregs for each tissue compartment was calculated to evaluate the balance of the immune environment in these compartments (Fig. 1A). There was a significantly higher CD8/Treg ratio in the normal bowel tissue compared with the tumor tissues in both primary and metastatic (*P* = 0.005 and *P* = 0.0046, respectively), whereas there was a significantly higher CD4eff/Treg ratio in PBMC compared with the tumor tissue, both primary and metastatic (*P* = 0.0073 and 0.0255, respectively). This suggests that the siNETs have a more inhibitory immune tumor microenvironment with lower effector/Treg ratio, whether primary or metastases, compared with normal tissue and PBMC.

### Single immune checkpoint molecules are expressed highly on Tregs and lower in PBMC in siNETs

T-cell subsets from normal tissue, PBMCs, and tumors from primary and metastatic sites were stained for checkpoint molecules and their expression analyzed by flow cytometry. To determine the balance of checkpoint molecule expression, cells were stained for co-inhibitory checkpoint molecules: CTLA-4, PD-1, TIM-3, and TIGIT and co-stimulatory checkpoint molecules: ICOS, 4–1BB, OX-40, and GITR. We also evaluated activation and proliferation markers, including Ki67, Granzyme B (GzmB), and CD25. Ki67 is an intracellular marker for proliferation, and GzmB is a serine protease most commonly found in cytotoxic T cells and natural killer cells (30), whereas CD25 is present on activated T cells (31).

For all checkpoint molecules, levels of expression on the Tregs were significantly higher than on the effector T cells (both CD8^+^ and CD4eff) for normal tissue, primary and metastatic tumor, and PBMCs (Fig. 1B; Supplementary Fig. S3). The only exception was TIM-3 and OX-40 expression on PBMCs for which there was no significant difference between the CD8^+^ or CD4eff, respectively, and the Tregs. For all of the other checkpoint molecules, there was a significant difference between both the effector T cells and Tregs in the PBMCs. We next explored whether there were differences between the single checkpoint molecule expression in the tissue types. PD-1 and CTLA-4 were significantly expressed on CD8^+^ cells in tumors compared with PBMC and normal tissues, whereas for CD8^+^ TIGIT was significantly higher in PBMC. TIM-3 and TIGIT were significantly expressed on CD4eff in metastatic tumors compared with the other tissue types, whereas PD-1 and CTLA-4 expressions were significantly lower in PBMC. PD-1, TIM-3, OX-40, and GITR expressions were significantly lower on Tregs in PBMC compared with the other tissue types (Supplementary Fig. S3). The activation landscape is also distinct in PBMC with significantly higher expression of GzmB on all T-cell subsets and CD25 on CD4eff compared with the other tissue types. There was no significant difference in single checkpoint expression between the tissue types across the T-cell subsets when split between patients who had SSA or not, except for TIGIT in CD8 normal tissue that was significantly higher in those who previously had SSA (Supplementary Fig. S5).

### Tumor-infiltrating lymphocytes in siNETs exhibit a more regulated co-expression phenotype compared with lymphocytes in normal tissue

**Figure 2.**
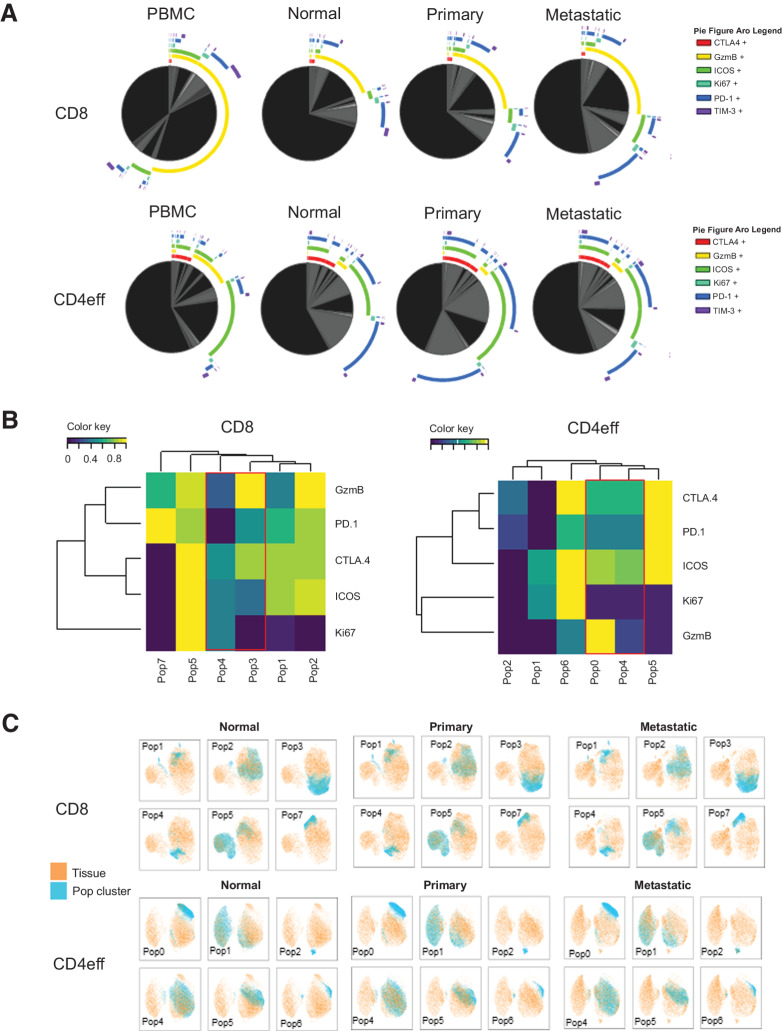
CD8^+^ in metastatic tissue is more regulated compared with other tissue types. Co-expressions of key B7 and TNFR superfamily co-inhibitory and co-stimulatory molecules on T-cell subsets were quantified by flow cytometry with matched PBMCs, normal tissue, and tumors from primary and metastatic sites. **A,** SPICE analysis of all CD8^+^ and CD4eff T cells displaying the mean co-expression of checkpoint molecules across tissue types. **B,** Unsupervised FlowSOM of CD8 and CD4eff clustering demonstrates that PBMCs (red box) are distinctly different to other tissues types. **C,** UMAP distribution of each FlowSOM CD8 and CD4eff population (blue) on each tissue subtype (orange).

To further characterize the immune microenvironment of siNETs, we compared the expression of multiple checkpoints on CD8^+^ and CD4eff T cells to interrogate the flow checkpoint phenotype overlap between the tissue types. The Treg population in siNETs was too small for any meaningful co-expression analysis and was, therefore, excluded in this analysis. The immune markers with the most co-expression data were used, including PD-1, CTLA-4, TIM-3, ICOS, GzmB, and Ki67. Data from all samples of each tissue type were obtained using hierarchical gating strategy (Boolean) on FlowJo and subsequently analyzed using a visualization software: “Simplified Presentation of Incredibly Complex Evaluations” (SPICE; ref. 32). We found a distinctive co-expression phenotype in PBMC compared with the other tissue types in both CD8^+^ and CD4eff T cells (Fig. 2A), whereas the differentiation of the immune landscape between normal tissue and tumor tissue was less apparent. The similarity in immune landscape between the normal tissue and that of the tumor was consistent across all of the samples obtained, suggesting that these expression patterns are representative of the tissue type, normal or tumor.

Unsupervised clustering of concatenated viable CD8^+^ and CD4eff T cells in PBMC, normal tissue, primary, and metastatic tumors revealed six T-cell subpopulations with 1,000 cell counts and more (Fig. 2B), with clusters highlighting PBMC (red box) in CD8^+^ (Pop3 and Pop4; Fig. 2B) and CD4eff (Pop0 and Pop4; Fig. 2B). Immune markers such as TIM-3 and 4–1BB with low expression on effector T cells were excluded in this analysis (Supplementary Fig. S4). The CD8^+^ PBMC cluster of Pop3 and Pop4 and CD4eff PBMC cluster of Pop0 and Pop4 (Fig. 2B) were distinctly different to the other tissue types with lower expression of checkpoint markers (PD-1, ICOS, and CTLA-4), suggesting a less regulated immune tumor environment in the periphery. The GzmB expressions on CD4eff PBMC cluster were high compared with tissue type, but varied on CD8. Visualization of the immune landscape by UMAP (33) dimension reduction likewise revealed these cluster populations (blue) overlaying on each tissue type (orange; Fig. 2C). The metastatic clusters Pop2 and Pop5 had highest expression of checkpoint markers on CD8, while on CD4eff, the clusters Pop1, Pop2, and Pop5 between the tissue types were similar. This mirrors the SPICE plots as depicted in Fig. 2A with increased checkpoint expression of PD-1, ICOS, and CTLA-4, whereas these checkpoint expressions were similar on CD4eff. This demonstrates that in siNETs, the CD8^+^ T cells are more regulated in metastatic tissue compared with other tissues and CD4eff.

**Figure 3.**
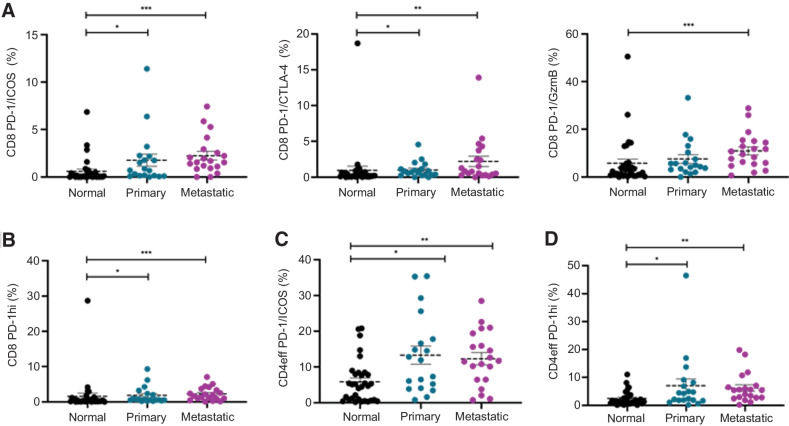
Effector T cells in metastatic tissue are significantly more regulated compared with other tissue types. Co-expressions of key B7 and TNFR superfamily coinhibitory and costimulatory molecules on T-cell subsets were quantified using PD-1 as backbone by flow cytometry with matched normal tissue and tumors from primary and metastatic sites. **A,** Graphs depict frequency of co-expression with PD-1 and ICOS, CTLA-4, and Granzyme B (GzmB) on CD8^+^. Horizontal bars represent the mean; error bars show ± standard error of the mean (SEM). **B,** Graphs depict frequency of high levels of PD-1 (PD-1hi) on CD8^+^. Horizontal bars represent the mean; error bars show ± SEM. **C,** Graphs depict frequency of co-expression with PD-1 and ICOS on CD4eff. Horizontal bars represent the mean; error bars show ± SEM. **D,** Graphs depict frequency of high levels of PD-1 (PD-1hi) on CD4eff. Horizontal bars represent the mean; error bars show ± SEM. *, *P* < 0.05; **, *P* < 0.005; ***, *P* < 0.0005.

In several solid tumors, combining CPIs, particularly the combination of PD-1 and CTLA-4 inhibition, has improved outcomes compared with PD-1 inhibition alone (8, 34–36), and many other checkpoint combination trials are underway (37). Because we found PD-1 to be highly expressed in the T-cell subsets of siNETs, we analyzed its co-expression with other checkpoint molecules to explore the rationale for combination therapy in this tumor type. PBMCs were excluded in these analyses as shown to be distinctly different to tissue. Using PD-1 as a backbone, we found that CD8^+^ tumor-infiltrating lymphocytes (TIL) had a significantly higher co-expression of PD-1/ICOS and PD-1/CTLA-4 (Fig. 3A) and high levels of PD-1 expression (PD-1hi) compared with normal tissue (Fig. 3B), suggesting a more regulated, chronically antigenic stimulated immune microenvironment in the tumor (38, 39). We also found CD8^+^ PD-1/GzmB was significantly higher in metastatic disease, demonstrating potentially terminally differentiated T cells in more advanced cancer (40). Similarly, we found a significantly higher CD4eff TILs co-expression of PD-1/ICOS (Fig. 3C) and PD-1hi (Fig. 3D) compared with normal tissue. There was no significant difference in these co-expression populations between the tissue types across the T-cell subsets when split between patients who had SSA or not, except for PD-1/ICOS in CD8 normal tissue that was significantly higher in those who previously did not have SSA (Supplementary Fig. S5). Taken together, our data may suggest that we could use PD-1 as a checkpoint immunomodulator backbone in combination with CTLA-4 or ICOS to treat patients with siNET, although this needs to be tested in the clinical setting.

### Evaluation of the geographical immune landscape shows that these tumors have poor infiltration by T cells

**Figure 4.**
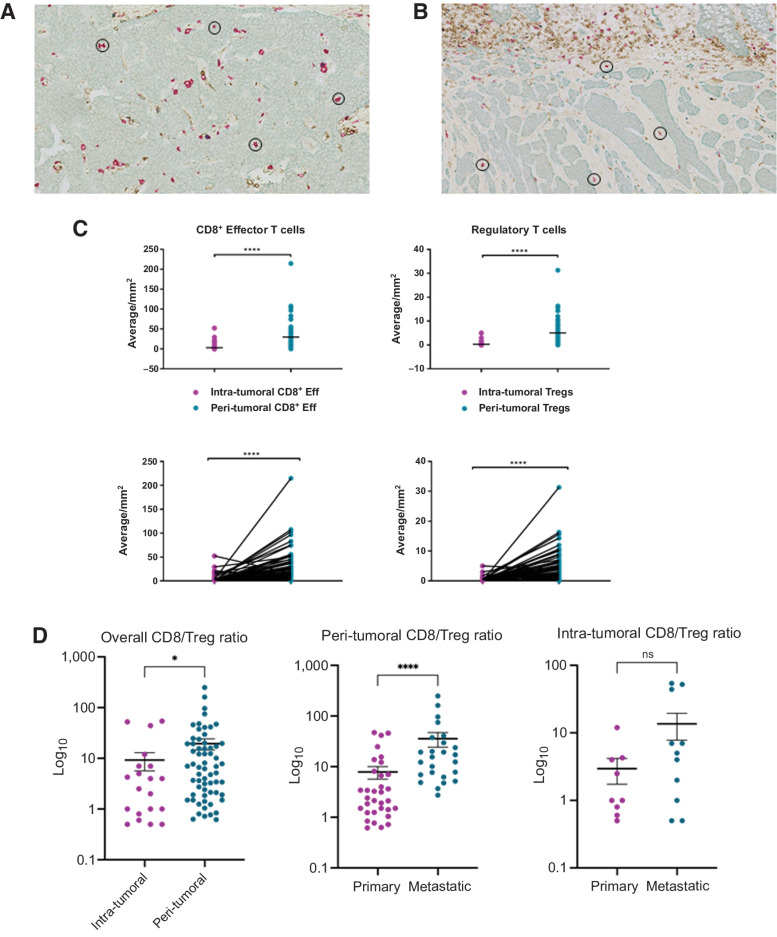
The geographical immune landscape in siNET shows predominantly peri-tumoral T cells. **A** and **B,** IHC staining of siNET. T-cell subsets stained are CD8^+^ T cells (red), CD4^+^ T cells (brown), and FoxP3^+^ (blue), and tumor cells are stained for Cytokeratin (green). These images of two siNET show examples of a tumor with intra-tumoral T cells (**A**) or with mainly peri-tumoral T cells (**B**). Examples of intra-tumoral or peri-tumoral T cells are circled in black in A and B, respectively. **C,** Plots of T-cell counts per mm^2^ showing that T cells are predominantly peri-tumoral rather than intra-tumoral in these tumors. **D,** Respective CD8/Treg ratio (Log_10_) in primary versus metastatic, peri-tumoral, and intra-tumoral. Horizontal bars represent the mean; error bars show ± standard error of the mean (SEM). *, *P* < 0.05; ****, *P* < 0.0001.

Despite providing detailed insight into the molecular immune environment of siNET, flow cytometry does not allow the geographical immune landscape to be defined, and for this reason we also undertook an IHC analysis. For all of the NET cases, FFPE tissue was obtained and stained using a multiplex IHC technique (23–25) to identify different T-cell subsets. Single-tissue sections were stained for cytokeratin, CD8^+^, CD4^+^, and FoxP3^+^. For this analysis, CD4^+^ T cells were not assessed as it was difficult to differentiate positive cells due to inadequate staining. The number of CD8^+^ and Tregs were determined per mm^2^ of tumor as described previously in the Methods (Fig. 4A, B, and D). Intra-tumoral T cells were defined as those fully in contact with tumor cells on all sides, whereas peri-tumoral T cells were not fully in contact with tumor and within 100 µm of the tumor edge. The analysis revealed that the majority of CD8^+^ and Treg T-cell subsets were peri-tumoral rather than intra-tumoral (Fig. 4) as had been observed. This analysis indicated that in fact only 10.1% CD8^+^ and 5.9% Tregs were intra-tumoral whereas 89.9% CD8^+^ and 94.1% Tregs were peri-tumoral in both primary and metastatic tumors (Fig. 4C).

For primary tumors (*n* = 34), 10.3% CD8^+^ and 3.2% Tregs were intra-tumoral whereas 89% CD8^+^ and 96.8% Tregs were peri-tumoral. Furthermore, there was a significantly higher CD8/Treg ratio in peri-tumoral compared with intra-tumoral (*P* = 0.018), suggesting that not only were there less intra-tumoral T cells, but the tumor microenvironment was more heavily regulated with lower intra-tumoral CD8/Treg ratio (Fig. 4D). For metastases (*n* = 28, comprising 25 mesenteric masses and 3 metastatic lymph nodes), 5.4% CD8^+^ and 5.7% Tregs were intra-tumoral whereas 94.6% CD8^+^ and 90.5% Tregs were peri-tumoral.

This indicates that there is a significantly lower number of intra-tumoral T cells compared with peri-tumoral in both primary and metastatic samples (*P* ≤ 0.001). No significant relationships were identified between the numbers of peri-tumoral CD8^+^ or Tregs and the clinical characteristics of the patient or tumor that we had collected (these included: gender, age, grade, Ki-67 index, metastasis, or if they were on treatment SSAs; Supplementary Table S1). However, interestingly, when we looked into CD8/Treg ratio in primary and metastatic sites, there was a significantly higher peri-tumoral CD8/Treg ratio in metastatic sites (*P* ≤ 0.0001) compared with primary sites, whereas there was no difference in intra-tumoral CD8/Treg ratio (*P* = 0.57). This is driven by a significantly high number of peri-tumoral Tregs in primary sites compared with metastases (*P* = 0.0006; Supplementary Fig. S6), and not peri-tumoral or intra-tumoral CD8s. The majority of our metastases were mesenteric metastases (69.4%), and with the mesentery being an organ that contains mainly fat, lymphatics, and blood vessels, this may explain why there was a significantly higher number of peri-tumoral lymphocytes in this population. In addition, there was one patient that had very high numbers of peri-tumoral T cells (both CD8^+^ and Treg). This was a female patient 77 years of age who had no known metastases and was not on treatment with SSAs (Patient # 25 in Supplementary Table S1). This might be a tumor that is attracting a larger than normal T-cell response but it is possible that this sample was taken from tumor that had invaded a lymph node that was not obvious by IHC but resulted in high numbers of T cells present around the tumor tissue.

### siNETs have a low tumor mutational burden compared with other tumor types in TCGA cohort

**Figure 5.**
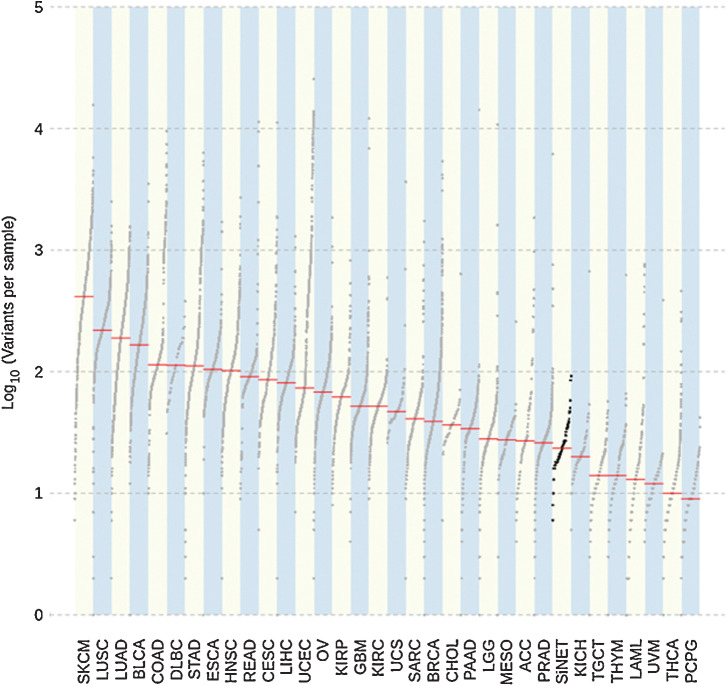
SiNET mutational load. This shows a comparison of the siNET mutational load against 33 TCGA cohorts from the Multicenter Mutation Calling in Multiple Cancers (MC3; ref. 63) project.

Tumor mutational burden (TMB), that is, the number of somatic mutations per DNA megabase, can be estimated from next-generation sequencing assays and used as a proxy for neoantigen burden. Elevated TMB has been observed to be predictive of improved survival in patients receiving immune CPIs across differing tumor types, although TMB thresholds vary between cancer subtypes (41–43). The mutational rate of siNET genomes is known to be low, with few recurrent aberrations (44). We confirmed that patients with siNET had a low TMB with a median of 0.5 mutations/Mb (range, 0.128–1.96). There was no correlation between the TMB and CD8/Treg ratio on IHC with a Spearman's Rho of −0.17 (95% confidence interval of −0.43 to 0.11; *P* = 0.22; Supplementary Fig. S8). We used the maftools package to compare the mutational burden of this dataset with TCGA cohorts and confirmed that TMB is low compared with other tumor types (Fig. 5), and similar to the mutational load previously described for siNETs (45–47).

*CDKN1b* is the most frequently reported recurrent mutation in siNET, present in approximately 8% of patients (47). In our cohort, *CDKN1B* mutations had a similar frequency with five patient samples (9%) identified (Supplementary Fig. S7). Interestingly, in one patient, NET45 (patient # 39 in Supplementary Table S1), the *CDKN1B* mutation was identified in both metastatic samples but not in the primary tumor.

## Discussion

Immunotherapy is currently being explored in many tumor types with encouraging results, but there has only been relatively limited investigation of this modality for patients with NET. In this article, we have characterized the immune-landscape in siNET with the aim of informing a rational approach for targeted immunotherapy in patients with NET.

We investigated the immune landscape in 40 patients with siNET. The T-cell subsets from the peripheral blood, primary tumor tissue, metastases, and normal small intestinal tissue were analyzed by multicolor flow cytometry. Analysis indicated that the primary tumors and metastatic tumors had a significantly lower ratio of CD8^+^ to Tregs than in normal bowel tissue, whereas there was a significantly lower ratio of CD4^+^ to Tregs than in blood. This signifies an imbalance between effector and Treg in a proliferating tumor, with a more inhibitory immune tumor microenvironment as compared with the normal bowel and suggests a potentially suppressive immune microenvironment within these tumors that may prevent tumor elimination.

Staining for co-inhibitory and co-stimulatory checkpoint molecules (CTLA-4, PD-1, TIM-3, TIGIT and ICOS, 4–1BB, OX-40, GITR, respectively) on the T-cell subsets showed that for all of the checkpoint molecules, their expression was highest on Tregs than in the other effector T-cell subsets (CD8^+^ and CD4eff), which is similar to what has been seen in other solid tumors (48, 49). This indicates that siNET might be uniquely targeted using immunomodulatory drugs directed toward depleting Tregs to shift the balance of the immune response in the tumors away from tumor tolerance and toward immune-mediated tumor elimination. Previous treatment with SSA did not seem to modulate the immune response in our cohort. We had one patient who had undergone PRRT that may have affected the immune tumor microenvironment. However, the number is too small to make any conclusion. We also identified that the immune checkpoint landscape had minimal flow phenotype overlap between PBMC and the rest of the tissue types. This is similar to previous published data (48) on flow cytometric analysis of PBMC and other tissue types.

High levels of PD-1 expression have previously been shown to identify tumor reactive T cells (38, 50, 51) and are associated with distinct transcriptomic, phenotypic, and functional properties (39, 52–54). Here, we demonstrate that these PD-1hi T cells were significantly more abundant in tumors compared with normal tissue, suggesting a chronically antigenic stimulated state. Interestingly, in one of the largest series of 64 siNETs, PD-1 expression on IHC was uncommon, with low levels of up to 1–20 cells/high power field that was limited to the stromal compartment (55), indicating the value of combining high-dimensional flow cytometry in evaluating the immune tumor microenvironment of siNET. The observation that PD-1 effector TILs were found to co-express multiple immune checkpoints, including CTLA-4 and ICOS suggest that combination therapy may be required to overcome resistance to monotherapy observed to date. Further functional experiments are needed to validate these findings and investigations exploring the therapeutic effect of these molecules, either alone or in conjunction with PD-1/PD-L1 blockade, is therefore warranted. Our data suggest the potential use of combination immune checkpoint antibodies with anti–PD-1 therapy as a backbone, which warrants further investigations in future clinical trials. A better understanding of expression patterns and cell surface density of target checkpoint receptors across T-cell subsets and tissue types is required to inform the most effective engineering of these antibodies and the identification of the best combinatory immunomodulating therapies. This is pertinent, given that immunomodulatory antibodies may require antibody-dependent cellular cytotoxicity to enhance Treg depletion, in addition to stimulating or blocking immune checkpoint molecules (49, 56).

IHC analysis of the tumors revealed that only 5%–10% of CD8^+^ and only 3–8% of Tregs are intra-tumoral whereas 89%–95% and 89%–97% are peri-tumoral, respectively. More specifically, there were significantly more peri-tumoral CD8/Tregs compared with intra-tumoral CD8/Tregs. This “excluded” phenotype suggests that the host immune system is able to mount a T-cell–mediated immune response intrinsically and/or peripherally and yet is able to escape these responses by hampering T-cell infiltration into the tumor microenvironment. There are several putative pathways that could explain this, including a possible role of extracellular matrix in promoting an immune exclusive phenotype, angiogenesis immune modulation via VEGFa, or oncogenic activation via WNT–β-catenin (57). Drugs targeting these pathways could be used in parallel with CPI to recruit these “excluded” T cells into the tumor microenvironment. It will be important in future studies to elucidate the mechanism of T-cell exclusion from these tumors to better understand this (55).

In this study, we have also shown that there was a high peri-tumoral CD8/Treg ratio in the metastatic sites. This is inverse to the published literature showing that there is a lower CD8/Treg ratio in metastatic site compared with primary site, likely due to immune evasion and escape, though in other tumor types like breast and colorectal cancers (58, 59). A plausible explanation may be due to the richness of lymphocytes in the mesentery due to lymphatics and vasculature, as the majority of our metastases were mesenteric. Moreover, it is well described in the literature that the gut has a distinct immune microenvironment with Treg cells playing a critical role due to the continued diverse antigen exposure (60). Finally, PD-L1 that is used as a predictive biomarker in some tumor types was not evaluated in our study and represents a potential limitation.

Previous groups have demonstrated a low TMB in siNET, with a median 0.1–1.098 variants per Mb (45–47, 61). TMB is an important determinant of clinical response to immune checkpoint blockade in most tumors (62) and, therefore, the low TMB found in siNETs may indicate less clinical benefit to immunotherapy with CPI. In addition, da Silva and colleagues (55) have shown a low IHC expression of PD-1 and PD-L1 in siNETs, together with low to moderate T-cell infiltration, suggesting that response to single PD-1 or PD-L1 inhibitors may be modest, although this remains to be investigated. Therefore, it is vital to enhance tumor killing by combining with another CPI or with an anticancer drug that targets siNETs to prime immunity by causing cell death and release of tumor antigens.

For targeted immunotherapy to be effective in patients with siNET, it is critical to understand the immune landscape in which they exist. This work gives an insight into the inhibitory nature of the immune environment in these tumors and indicates that T cells are largely absent from within the tumor mass in siNET. It will be of great interest in future work to understand the reasons for the absence of T cells, primarily if there is an active exclusion mechanism at play or if the tumors are lacking sufficient neoantigens for the endogenous immune system to recognize the threat and act to eliminate them. We have, however, shown that there are potential immune-modulatory targets for siNETs in the form of high checkpoint molecule expression on Tregs that could provide a strategy for therapeutic intervention to tip the balance of the immune response to these tumors away from tumor tolerance and toward tumor elimination.

## Supplementary Material

Supplementary Figure

Supplementary Figure

Supplementary Figure

Supplementary Figure

Supplementary Figure

Supplementary Figure

Supplementary Figure

Supplementary Figure

Supplementary Data

Supplementary Table
