# Supplementary figures and images for: Systematic Evaluation of the Immune Environment of Small Intestinal Neuroendocrine Tumors
Source: Clin Cancer Res. 2022 Mar 23;28(12):2657–68. doi: 10.1158/1078-0432.CCR-21-4203 (PMC9359734; doi:10.1158/1078-0432.CCR-21-4203)

## Slide 1
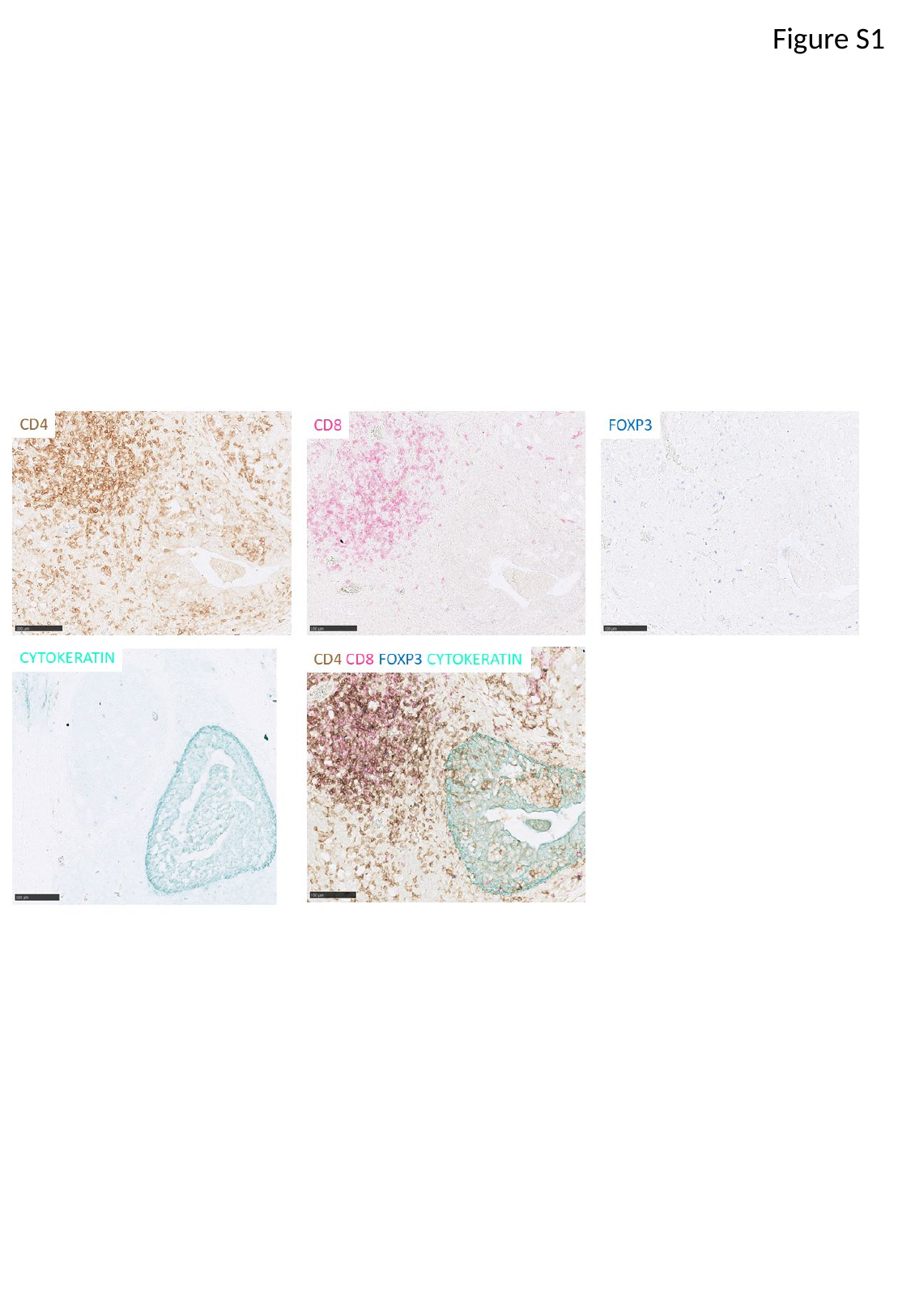

Figure S1

Supplement: Supplementary Figure [file ccr-21-4203_figure_s1_suppfs1.pptx]

## Slide 1
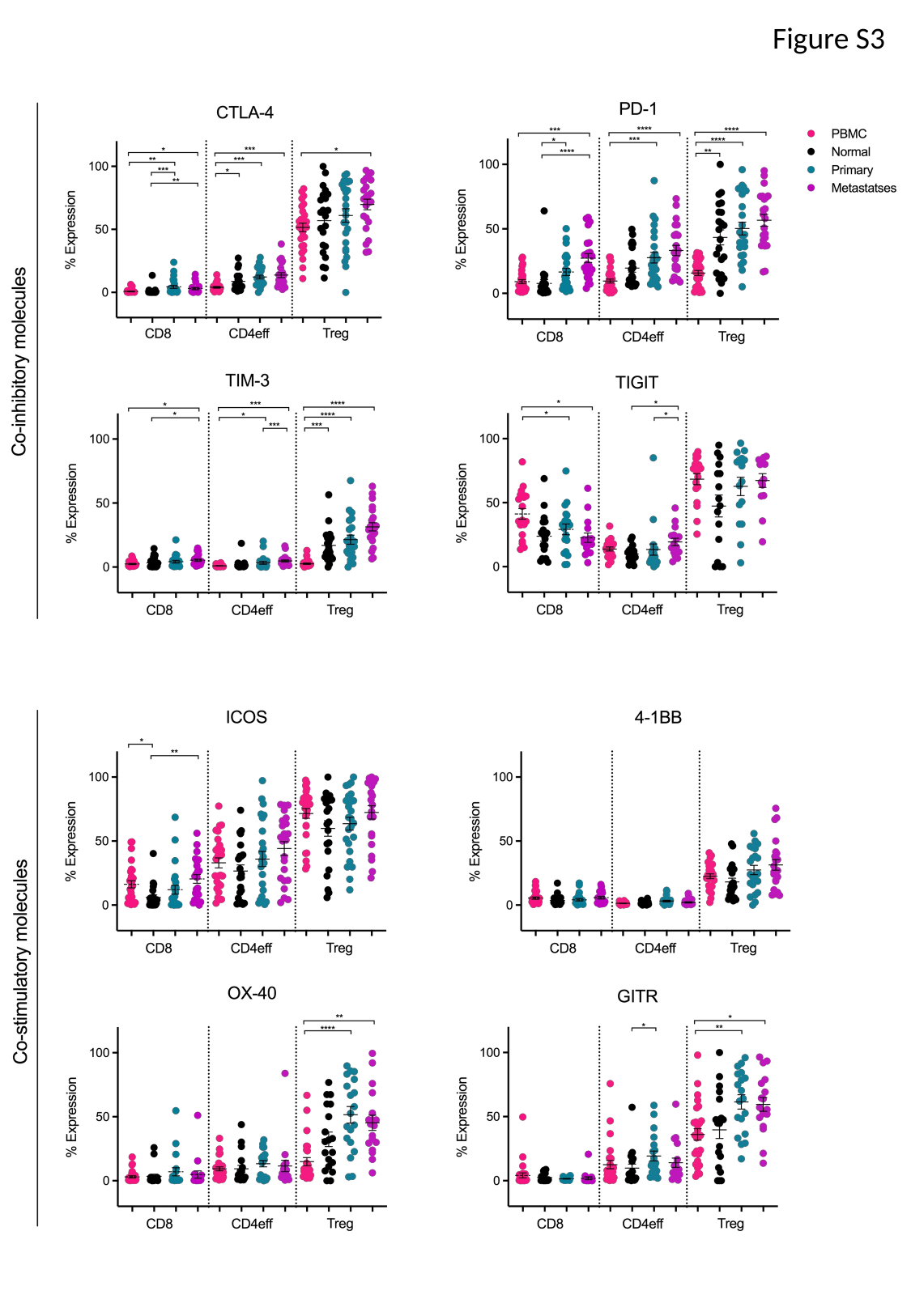

Figure S3

Supplement: Supplementary Figure [file ccr-21-4203_figure_s3_suppfs3.pptx]

## Slide 1
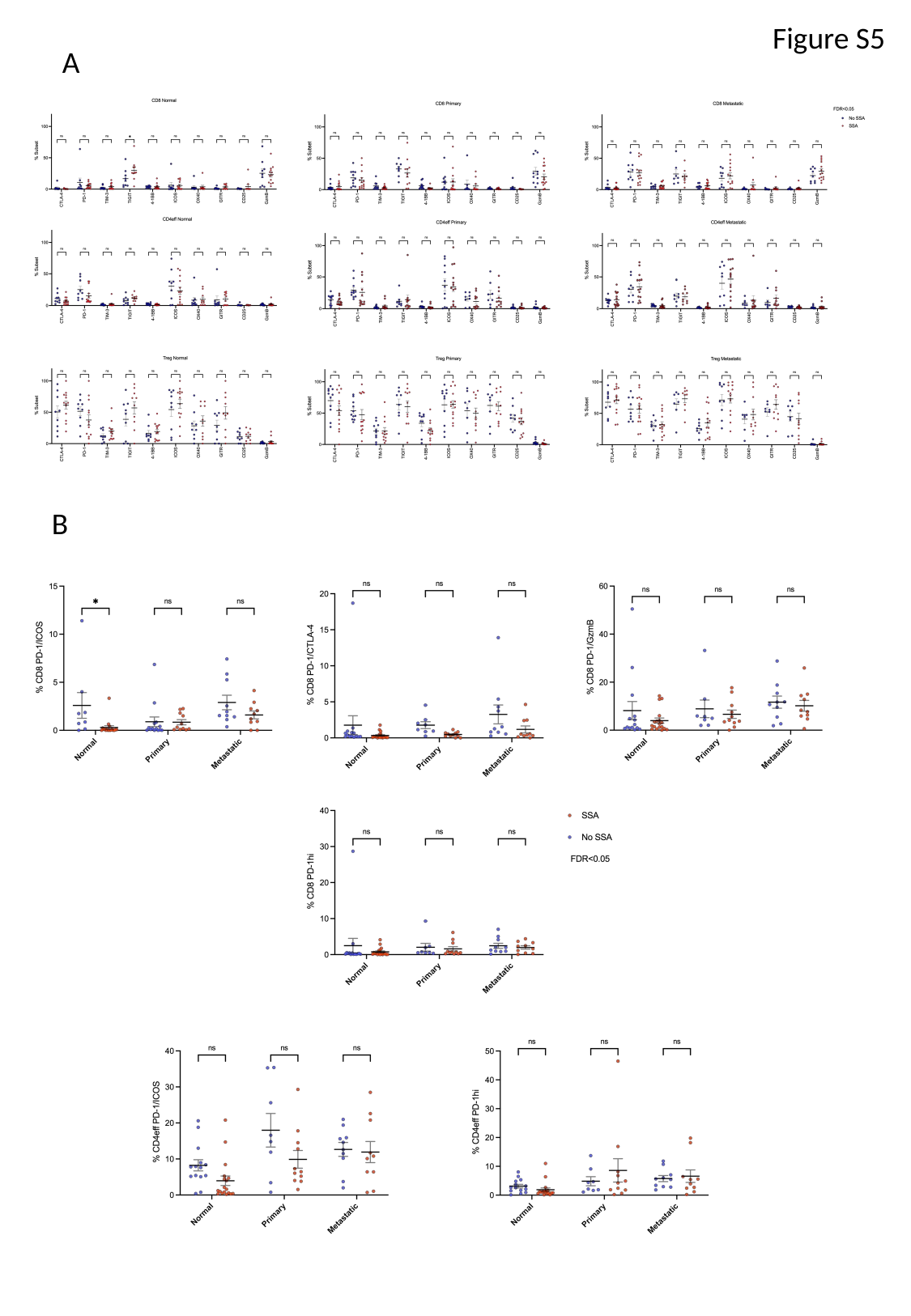

Figure S5
A
B

Supplement: Supplementary Figure [file ccr-21-4203_figure_s5_suppfs5.pptx]

## Slide 1
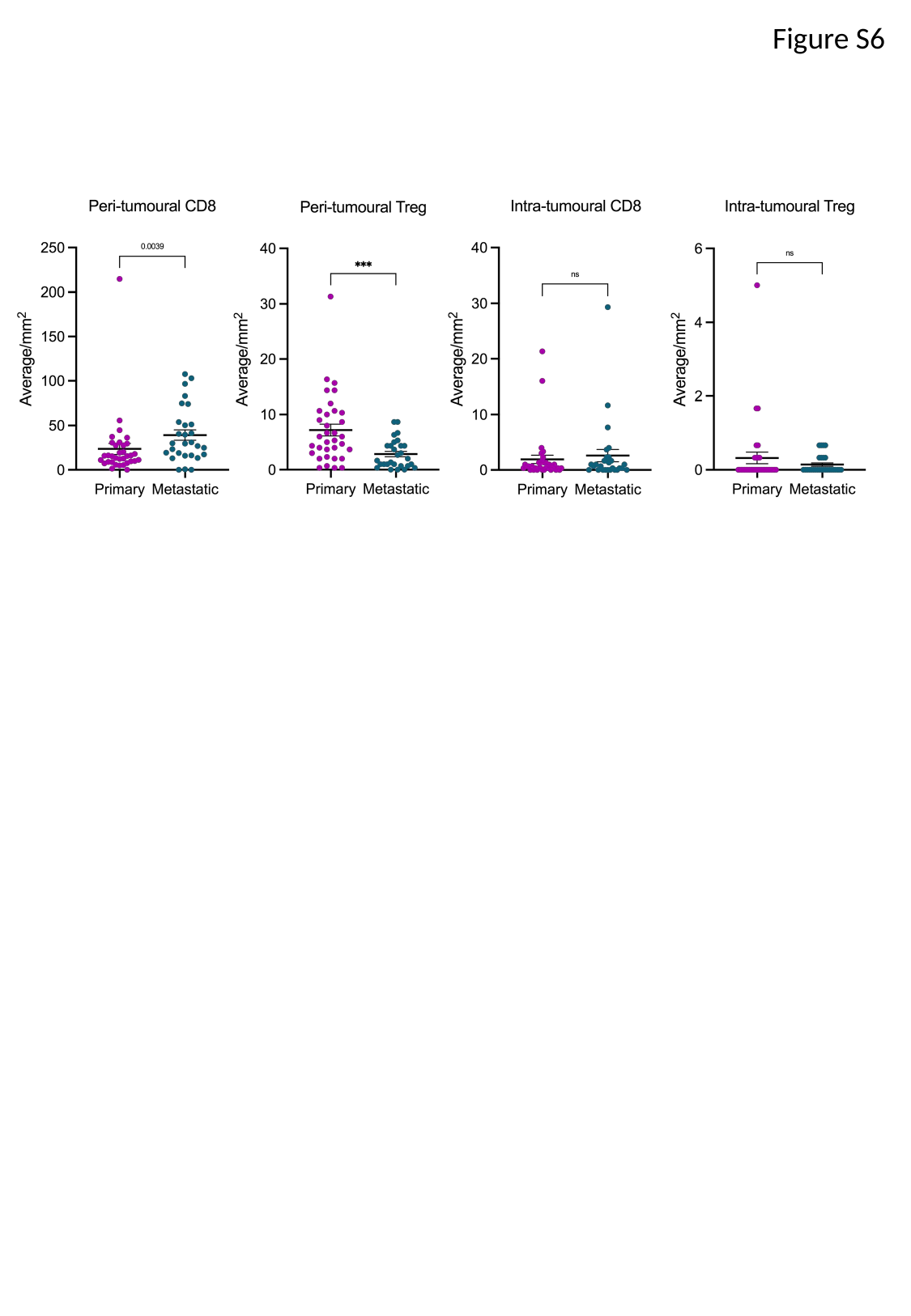

Figure S6

Supplement: Supplementary Figure [file ccr-21-4203_figure_s6_suppfs6.pptx]

## Slide 1
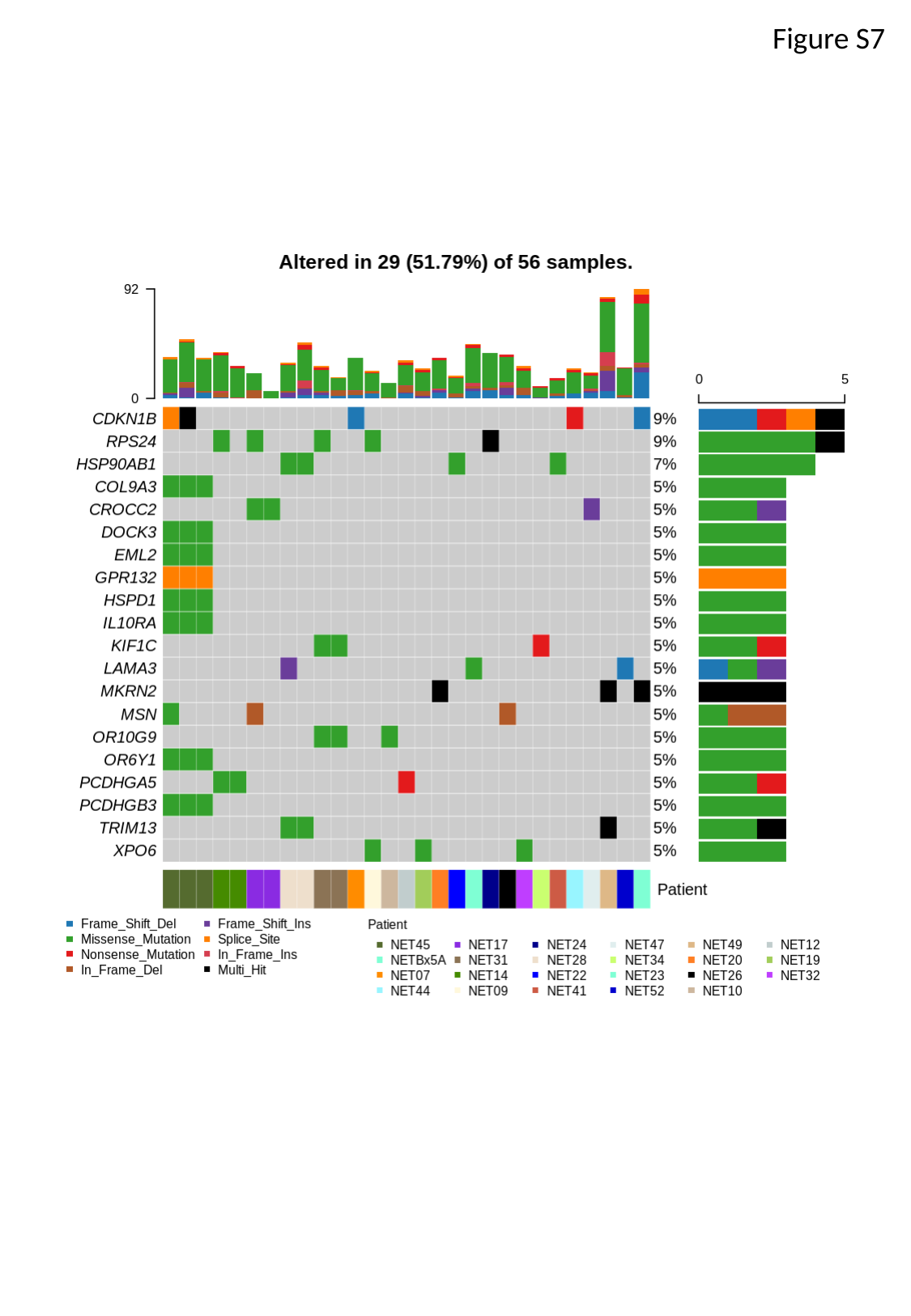

Figure S7

Supplement: Supplementary Figure [file ccr-21-4203_figure_s7_suppfs7.pptx]

## Slide 1
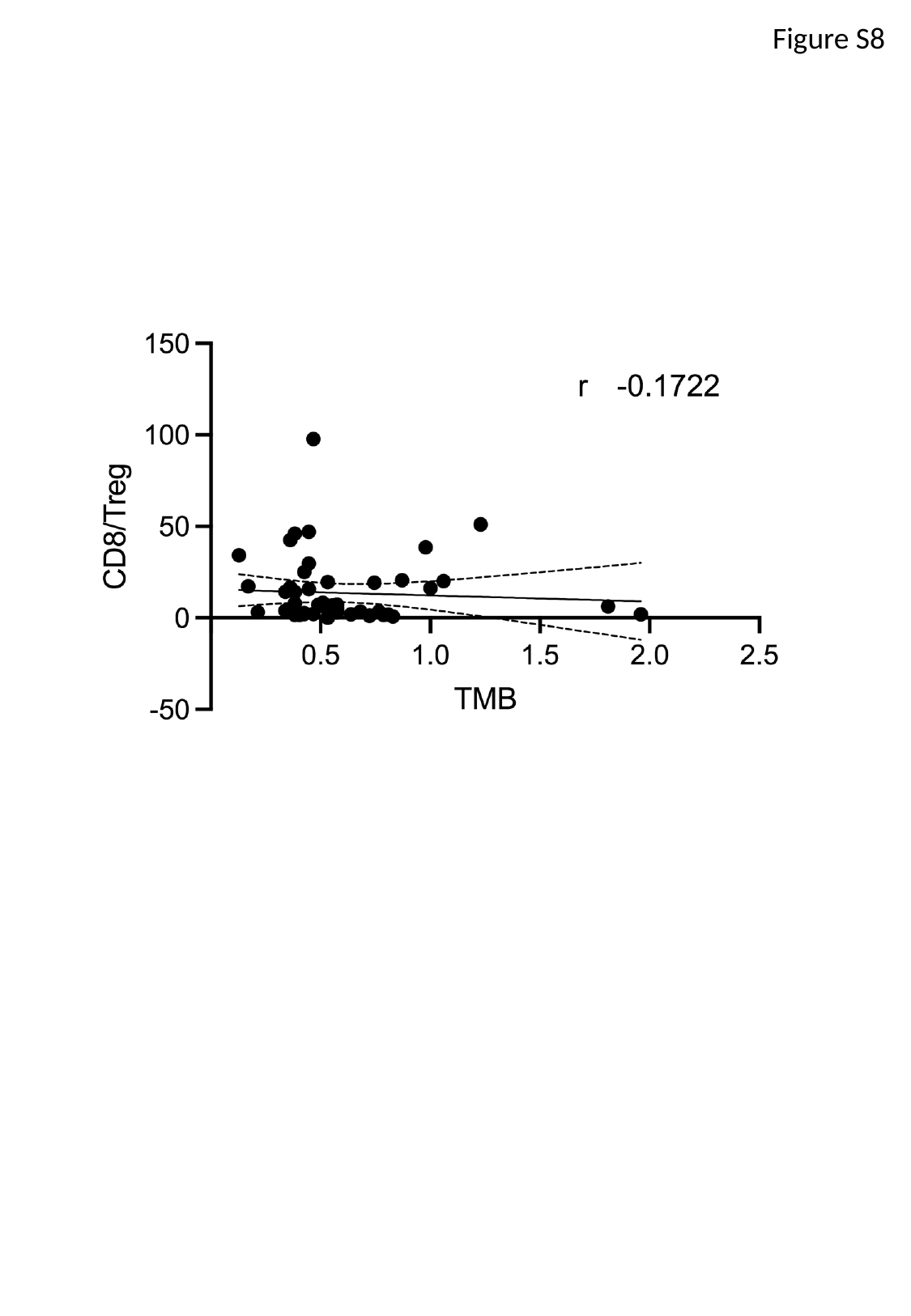

Figure S8

Supplement: Supplementary Figure [file ccr-21-4203_figure_s8_suppfs8.pptx]
