# Supplementary material for: Systematic Evaluation of the Immune Environment of Small Intestinal Neuroendocrine Tumors
Source: Clin Cancer Res. 2022 Mar 23;28(12):2657–68. doi: 10.1158/1078-0432.CCR-21-4203 (PMC9359734; doi:10.1158/1078-0432.CCR-21-4203)
Supplement: Supplementary Figure [file ccr-21-4203_figure_s2_suppfs2.pptx]

## Slide 1
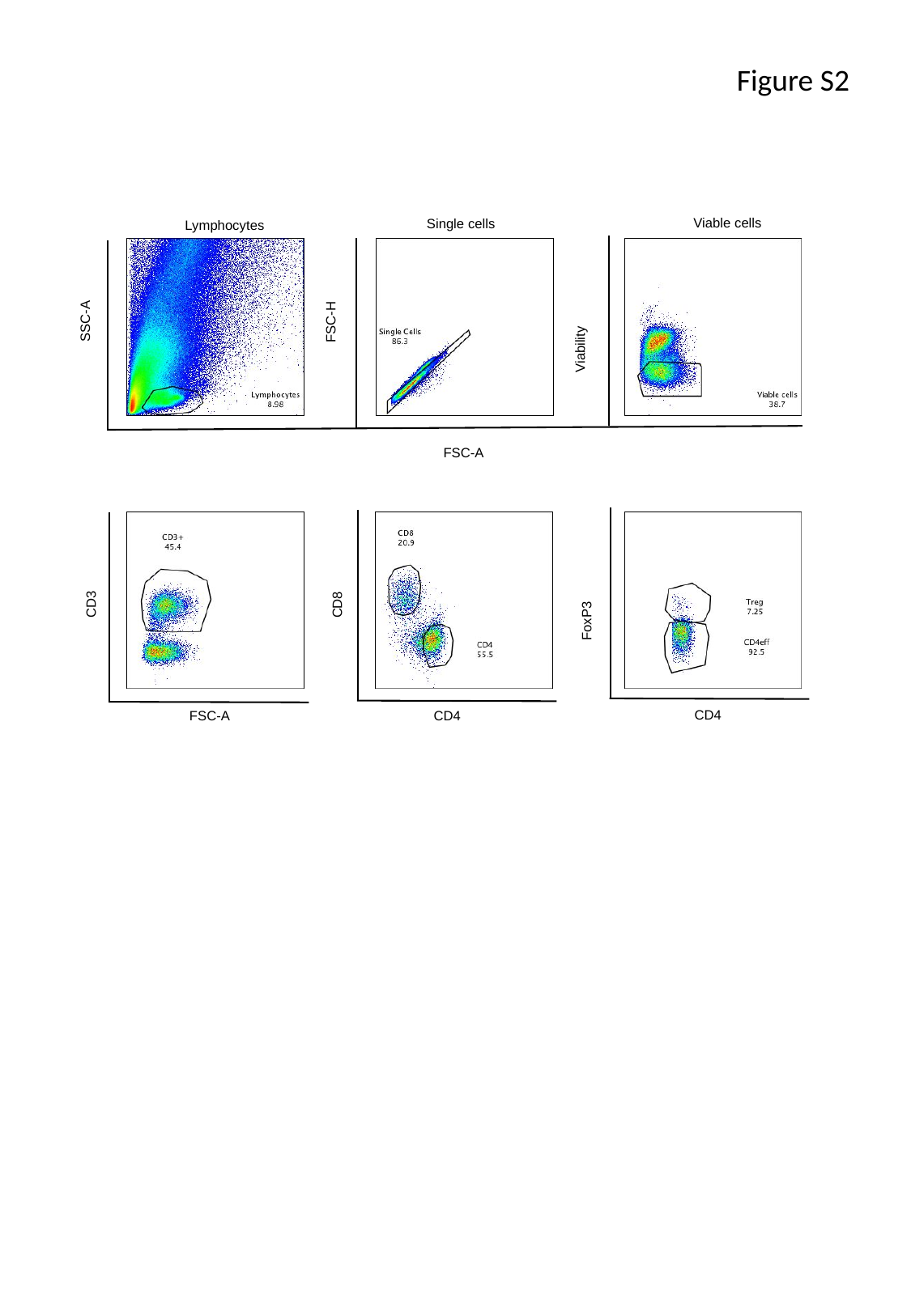

Figure S2
Viable cells
Single cells
Lymphocytes
SSC-A
FSC-H
Viability
FSC-A
CD8
CD3
FoxP3
CD4
FSC-A
CD4
