# Supplementary material for: Systematic Evaluation of the Immune Environment of Small Intestinal Neuroendocrine Tumors
Source: Clin Cancer Res. 2022 Mar 23;28(12):2657–68. doi: 10.1158/1078-0432.CCR-21-4203 (PMC9359734; doi:10.1158/1078-0432.CCR-21-4203)
Supplement: Supplementary Figure [file ccr-21-4203_figure_s4_suppfs4.pptx]

## Slide 1
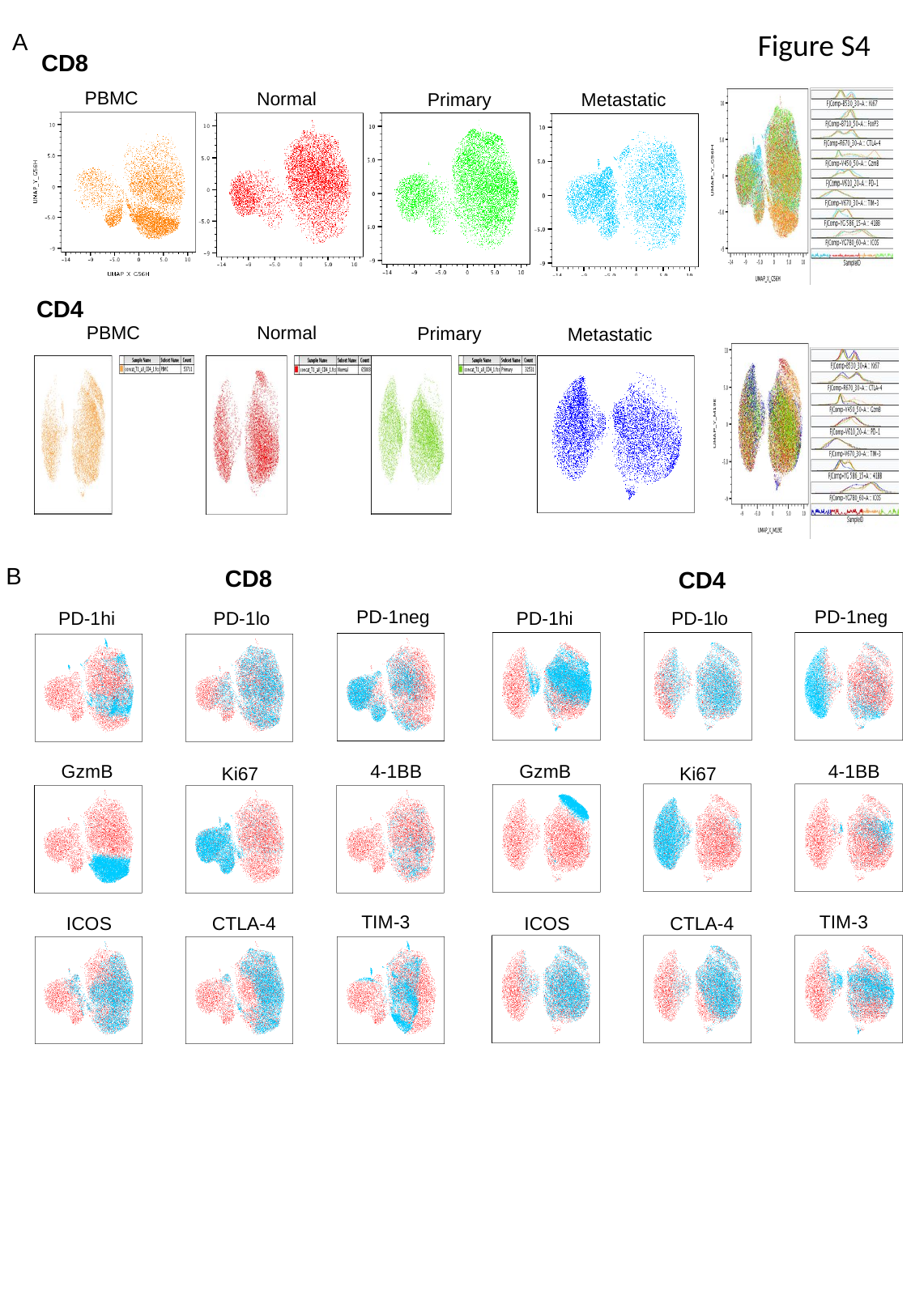

Figure S4
A
CD8
PBMC
Normal
Metastatic
Primary
CD4
PBMC
Normal
Primary
Metastatic
B
CD8
CD4
PD-1neg
PD-1hi
PD-1lo
4-1BB
GzmB
Ki67
TIM-3
CTLA-4
ICOS
PD-1neg
PD-1hi
PD-1lo
4-1BB
GzmB
Ki67
TIM-3
CTLA-4
ICOS
