# Supplementary material for: Systematic Evaluation of the Immune Environment of Small Intestinal Neuroendocrine Tumors
Source: Clin Cancer Res. 2022 Mar 23;28(12):2657–68. doi: 10.1158/1078-0432.CCR-21-4203 (PMC9359734; doi:10.1158/1078-0432.CCR-21-4203)
Supplement: Supplementary Data [file ccr-21-4203_supplementary_legend_suppsl.docx]

**Supplementary figure legends**

**Figure S1:** Single staining for CD4, CD8, FOXP3, Cytokeratin and multiplex staining including all four.

**Figure S2: Gating strategy**

**Figure S3: Single checkpoint expression on T cell subsets**

The percentage expression of immune checkpoint molecules on different T cell subsets of PBMCs, normal tissue, primary tumours and metastases (mesenteric masses and lymph nodes) as assessed by flow cytometry is shown. The median values for each subset are indicated by horizontal black bars. Checkpoint molecule expression tended to be higher on the regulatory T cells.

**Figure S4: Multiparametric analysis of CD8 and CD4**

(A) Distinct distribution of CD8 and CD4 checkpoint and proliferation markers across tissue type

(B) Checkpoint distribution on UMAP on concatenated CD8 and CD4. Checkpoint expression marked in blue and negative expression marked in red.

**Figure S5: Single checkpoint and co-expression checkpoint expression showed minimal difference between patients who had previous somatostatin analogues (SSA) or no SSA.** (A) Graph depicts the T cell subsets of CD8, CD4eff and Treg in the tumour microenvironment only (normal tissue, primary tissue and metastases. Statistical analyses using 2-way ANOVA and corrected false discovery rate of <0.05. *, P < 0.05.

(B) Graph depicts the co-expression data of CD8 and CD4eff in the tumour microenvironment only (normal tissue, primary tissue and metastases. Statistical analyses using 2-way ANOVA and corrected false discovery rate of <0.05. *, P < 0.05.

**Figure S6. Raw values of respective peri-tumoural and intra-tumoural CD8 and Treg average count per mm^2^.**

Respective raw values of CD8 and Treg average count per mm^2^ in primary vs metastatic, peri-tumoural and intra-tumoural. Horizontal bars represent the mean; error bars show ± standard error of the mean (SEM). *, P < 0.05; ****, P <0.0001.

**Figure S7. Mutational profile of sequenced siNET samples**

Figure shows an oncoplot with the top-20 most frequently mutated genes in the siNET cohort. *CDKN1B* is mutated in 5 out of the 56 (9%) sequenced samples.

**Figure S8. There was no correlation between tumour mutational burden (TMB) and CD8/Treg ratio on immunohistochemistry.** Figure shows a correlation plot between TMB and CD8/Treg ratio.
