# Supplementary material for: Systematic Evaluation of the Immune Environment of Small Intestinal Neuroendocrine Tumors
Source: Clin Cancer Res. 2022 Mar 23;28(12):2657–68. doi: 10.1158/1078-0432.CCR-21-4203 (PMC9359734; doi:10.1158/1078-0432.CCR-21-4203)
Supplement: Supplementary Table [file ccr-21-4203_supplementary_table_1_suppts1.docx]

**Table S1. Data collected from patient cohort.**

| ***ID*** | ***NET ID*** |  | ***Sex*** | ***Age*** | ***Grade*** | ***Ki67*** | ***Metastasis*** | ***Treatment**** |
| --- | --- | --- | --- | --- | --- | --- | --- | --- |
| 1 | NET03 |  | Male | 66 | 1 | 2 | No | Nil |
| 2 | NET05 |  | Male | 48 | 1 | 2 | Yes | SSA |
| 3 | NET07 |  | Female | 51 | 1 | 2 | Yes | SSA |
| 4 | NET09 |  | Male | 76 | 1 | <2 | No | SSA |
| 5 | NET10 |  | Female | 64 | 1 | 1 | Yes | SSA |
| 6 | NET12 |  | Female | 73 | 1 | <2 | Yes | SSA |
| 7 | NET13 |  | Male | 47 | 1 | <2 | Yes | SSA |
| 8 | NET17 |  | Female | 49 | 1 | <2 | Yes | Nil |
| 9 | NET18 |  | Male | 76 | 1 | <2 | Yes | SSA |
| 10 | NET19 |  | Female | 60 | 1 | <1 | No | Nil |
| 11 | NET22 |  | Female | 33 | 1 | <2 | Yes | SSA |
| 12 | NET24 |  | Male | 85 | 1 | <2 | Yes | SSA |
| 13 | NET25 |  | Male | 75 | 1 | <2 | Yes | SSA |
| 14 | NET27 |  | Male | 55 | 1 | <2 | Yes | SSA |
| 15 | NET29 |  | Male | 52 | 1 | <2 | Yes | Nil |
| 32 | NET32 |  | Male | 61 | 1 | <2 | Yes | SSA |
| 17 | NET33 |  | Male | 47 | 1 | <2 | No | SSA |
| 18 | NET34 |  | Male | 69 | 1 | <2 | No | SSA |
| 19 | NET35 |  | Male | 57 | 1 | <2 | Yes | SSA |
| 20 | NET40 |  | Male | 27 | 1 | <2 | Yes | Nil |
| 21 | NET41 |  | Male | 42 | 1 | <2 | Yes | SSA |
| 22 | NET43 |  | Female | 71 | 1 | <2 | Yes | SSA |
| 23 | NET47 |  | Female | 60 | 1 | <2 | Yes | Nil |
| 24 | NET49 |  | Female | 58 | 1 | <1 | Yes | SSA |
| 25 | NET51 |  | Female | 77 | 1 | <2 | No | Nil |
| 26 | NET52 |  | Female | 65 | 1 | <1 | Yes | SSA |
| 27 | NET08 |  | Male | 55 | 2 | 4 | Yes | SSA |
| 28 | NET14 |  | Female | 62 | 2 | 5 | Yes | SSA |
| 29 | NET15 |  | Female | 58 | 2 | 3 | Yes | Nil |
| 30 | NET16 |  | Female | 67 | 2 | 5 | No | Nil |
| 31 | NET20 |  | Male | 75 | 2 | 3 | Yes | SSA |
| 32 | NET23 |  | Male | 78 | 2 | 3 | Yes | Nil |
| 33 | NET26 |  | Male | 45 | 2 | 6 | No | Nil |
| 34 | NET28 |  | Male | 63 | 2 | 4 | No | Nil |
| 35 | NET30 |  | Female | 70 | 2 | 20 | Yes | Nil |
| 36 | NET31 |  | Male | 62 | 2 | 3 | Yes | SSA |
| 37 | NET38 |  | Male | 49 | 2 | 4 | No | Nil |
| 38 | NET44 |  | Female | 67 | 2 | 3 | Yes | SSA |
| 39 | NET45 |  | Male | 63 | 2 | 4 | Yes | Nil |
| 40 | NETBx5 |  | Female | 69 | 3 | 40 | Yes | PRRT |

*SSA = Somatostatin analogues, Nil = Patient not on any treatment prior to sample collection, PRRT = Peptide Receptor Radionuclide Therapy
